# Supplementary material for: Evaluation of CDK9 Inhibition by Dinaciclib in Combination with Apoptosis Modulating izTRAIL for the Treatment of Colorectal Cancer
Source: Biomedicines. 2023 Mar 16;11(3):928. doi: 10.3390/biomedicines11030928 (PMC10045754; doi:10.3390/biomedicines11030928)
Supplement: Supplementary file 1 [file biomedicines-11-00928-s001.zip › biomedicines-2084672-supplementary.pdf]

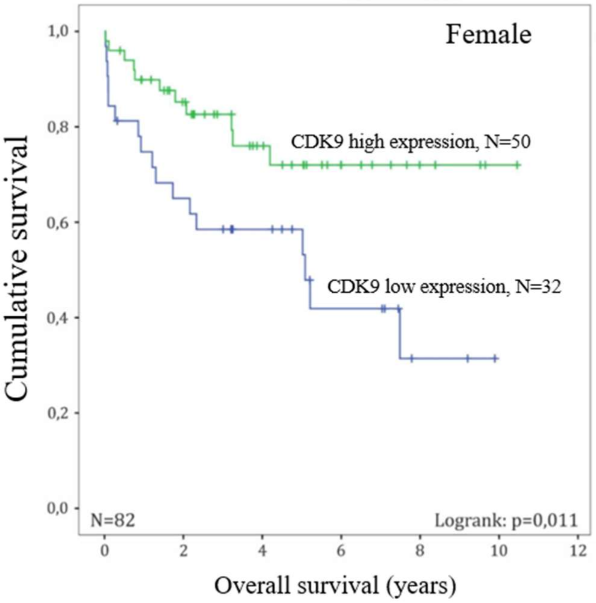

Figure S1: Survival time analysis of female patients depending on the level of CDK9 expression in colorectal tumor tissues.

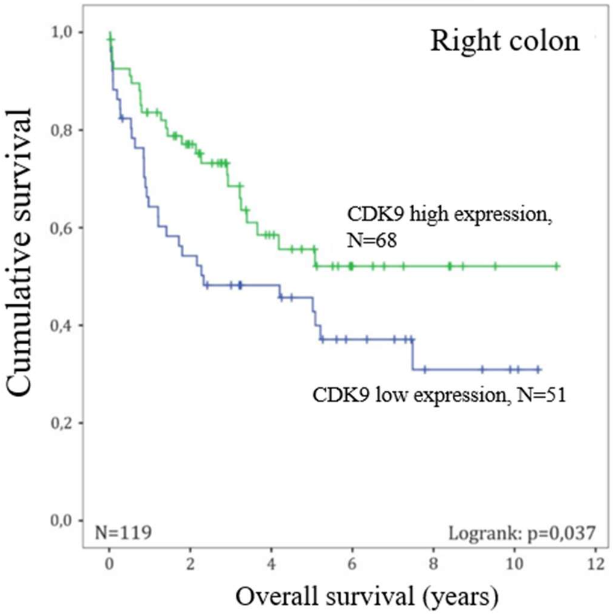

Figure S2: Survival time analysis of patients with right-sided colon cancer depending on the level of CDK9 expression in tumor tissues.

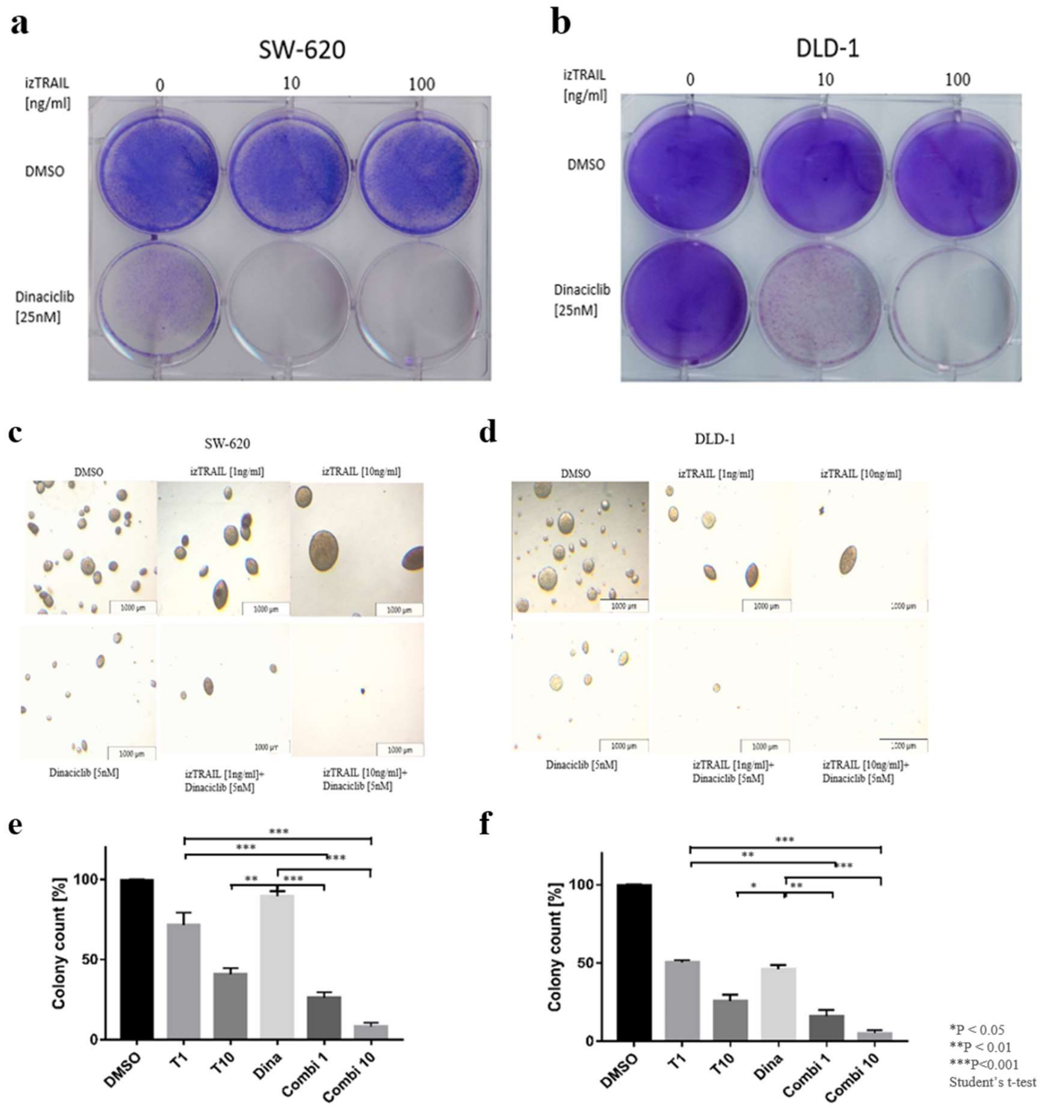

Figure S3: Synergistic effect of Dinaciclib treatment in combination with izTRAIL in SW-620 and DLD-1 cells. **a** and **b** 50,000 cells/well of SW-620 and DLD-1 cells seeded into 6-well plates and were treated with solvent (DMSO) or Dinaciclib (25 nM) for 1 h and subsequently stimulated with izTRAIL for 24 h. Long-term survival was visualized after 7 days by crystal violet staining. **c** SW-620 (5000 cells/well) and **d** HT-29 (2000 cells/well) were seeded into 6-well plates in soft agar supplemented with solvent (DMSO), izTRAIL (1, 10 ng/mL), and Dinaciclib (5 nM), respectively, alone or in combination. Formed colonies were photographed for three times in bright field at specific positions located on each fan-shaped center of every well divided into three equal parts and subsequentially visualized and quantified using MTT staining after 21–28 days. One of five independent experiments is shown. **e** and **f** All values are presented as means ± SD. * p <0.05, ** p <0.01, *** p <0.001; Student's t-test.

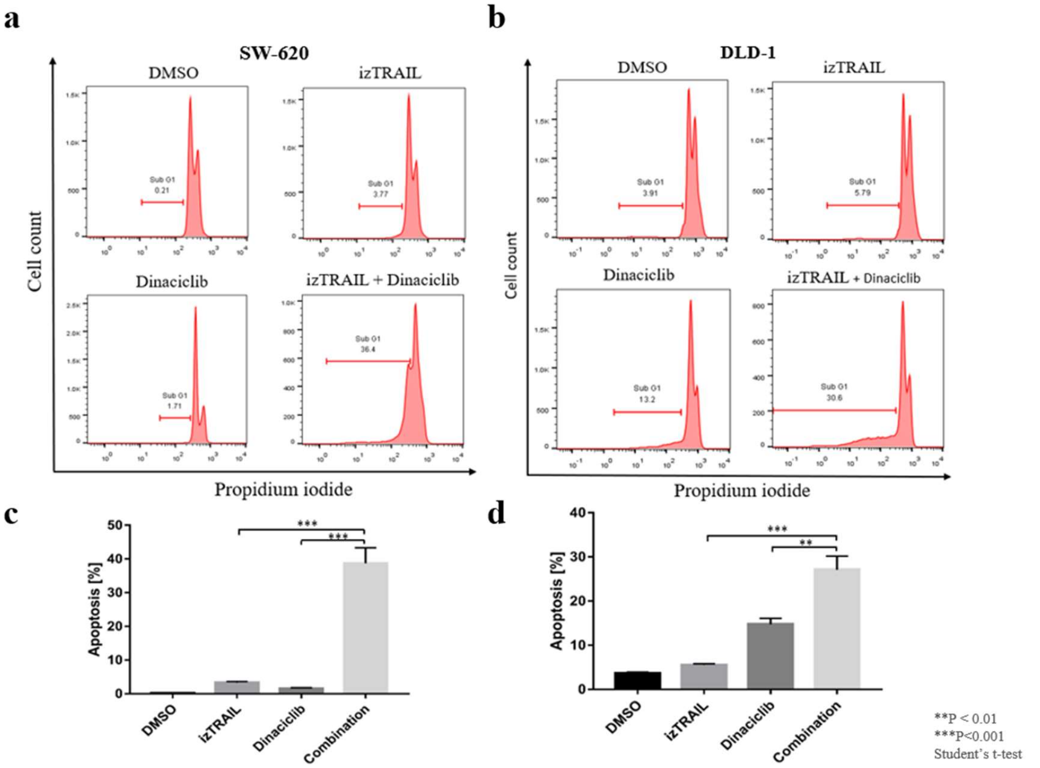

Figure S4: Changes in Sub G1 phase of cell cycle and apoptosis induction in SW-620 and DLD-1 cells under the influence of pharmacological CDK9 inhibition plus izTRAIL. **a** and **b** Analysis of cell cycle Sub G1 phase by (propidium iodide) PI staining for HT-29 cells. Flow cytometry was used to assess Sub G1 DNA content in the entire cell population. Untreated cells were used as negative control (DMSO), cells were treated either with TRAIL (10 ng/ml) and Dinaciclib (25 nM) or the combination for 24 h. For each treatment condition, 10,000 events were recorded and cellular debris was excluded from the analysis. **c** and **d** Data are presented as means ± SD; ** $p < 0.01$, *** $p < 0.001$; Student's *t*-test.

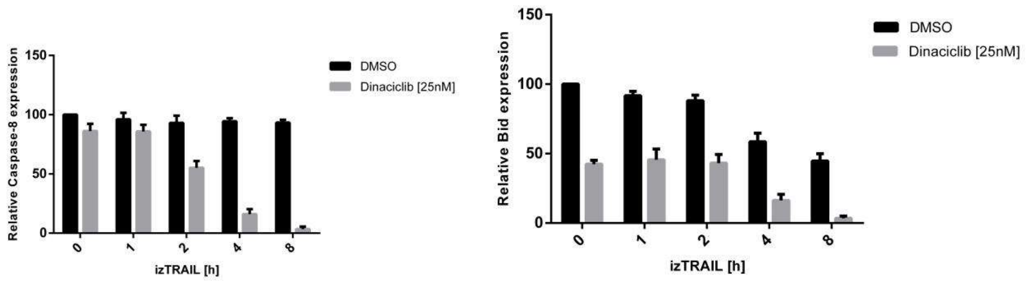

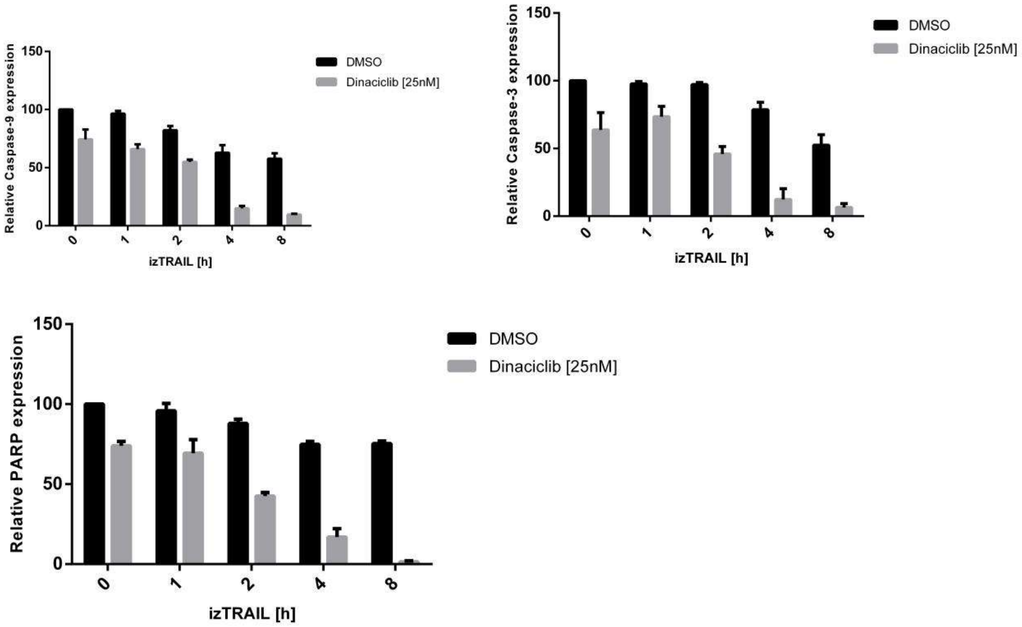

Figure S5: Western-Blot quantification of apoptosis-related protein expression. HT-29 cells were preincubated with DMSO or Dinaciclib (25nM) for 12 h and subsequently stimulated with izTRAIL (10 ng/mL) for the indicated periods (0, 1, 2, 4, 8 h). Cells were lysed and subjected to western blotting at least three times. Data is shown as % of untreated cells at 0 h.

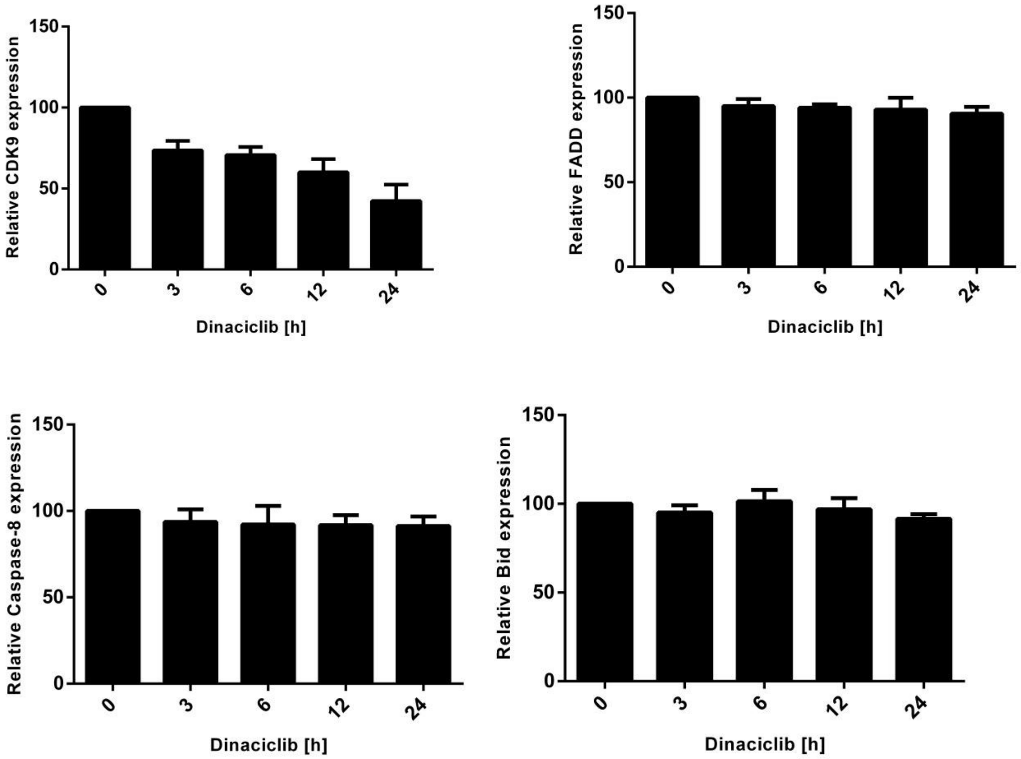

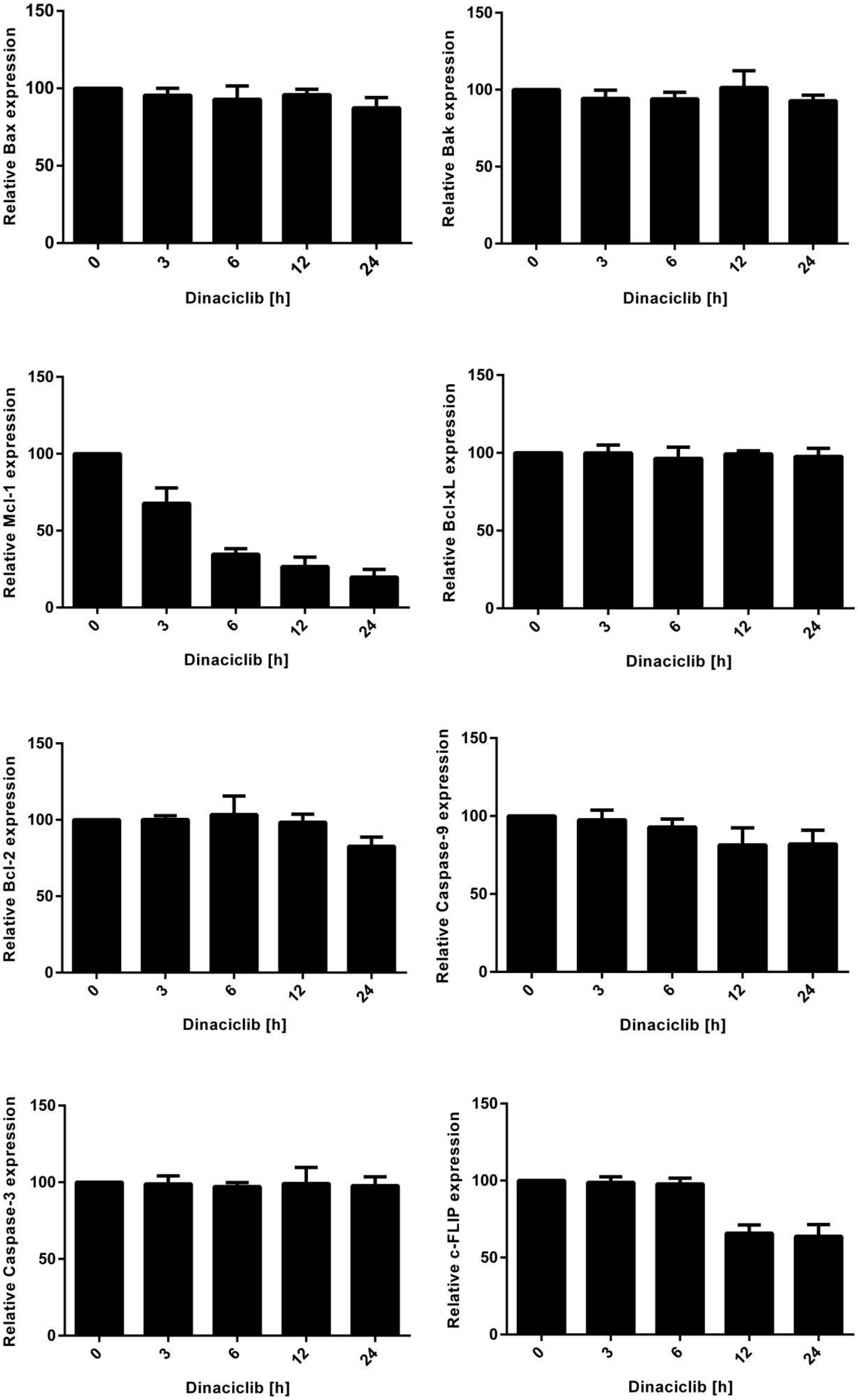

Relative Bax expression
150
100
50
0
0
3
6
12
24
Dinaciclib [h]
Relative Bak expression
150
100
50
0
0
3
6
12
24
Dinaciclib [h]
Relative Mcl-1 expression
150
100
50
0
0
3
6
12
24
Dinaciclib [h]
Relative Bcl-xL expression
150
100
50
0
0
3
6
12
24
Dinaciclib [h]
Relative Bcl-2 expression
150
100
50
0
0
3
6
12
24
Dinaciclib [h]
Relative Caspase-9 expression
150
100
50
0
0
3
6
12
24
Dinaciclib [h]
Relative Caspase-3 expression
150
100
50
0
0
3
6
12
24
Dinaciclib [h]
Relative c-FLIP expression
150
100
50
0
0
3
6
12
24
Dinaciclib [h]

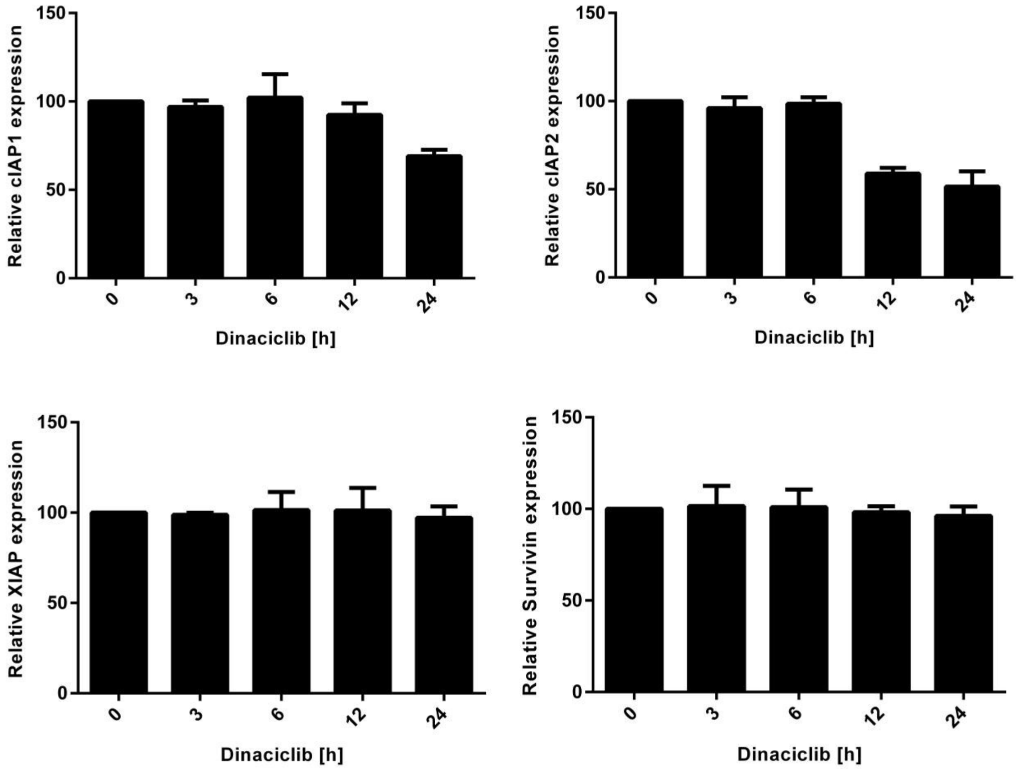

Figure S6: Western-Blot quantification of apoptosis-related protein expression in HT-29 after treatment with 25 nM Dinaciclib for the indicated periods.

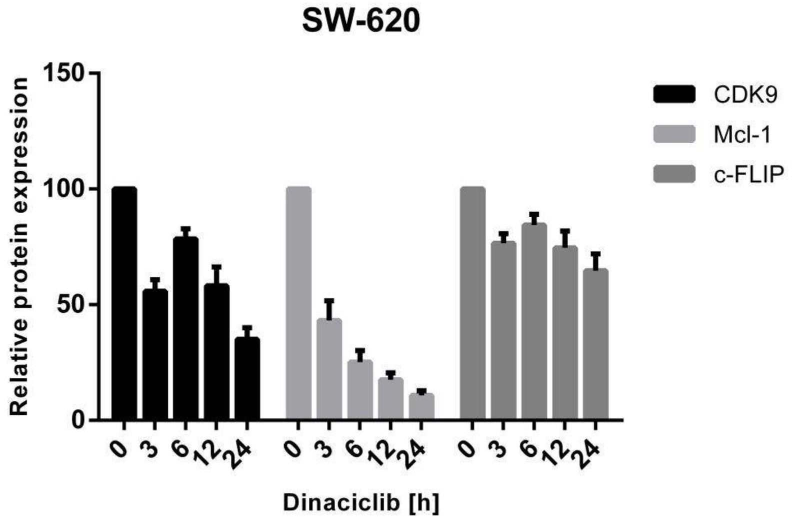

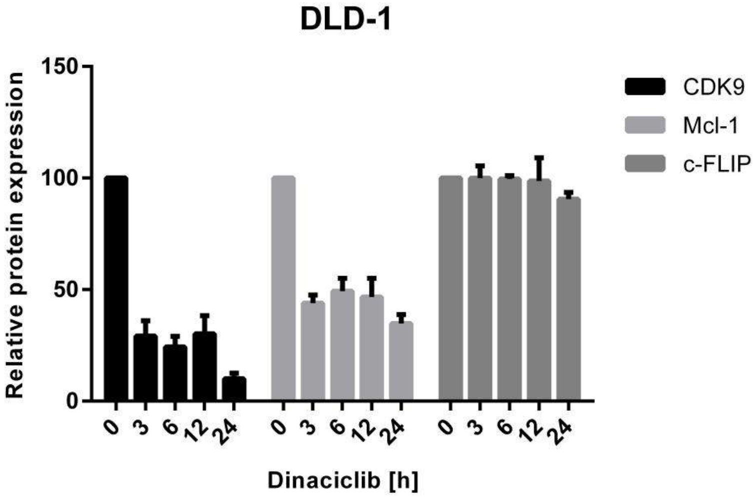

Figure S7: Western-Blot quantification of CDK-9, Mcl-1 and c-FLIP expression in SW-620 and DLD-1. Cells were treated with 25 nM Dinaciclib for the indicated periods.
